# A new look at risk patterns related to coronary heart disease incidence using survival tree analysis: 12 Years Longitudinal Study

**DOI:** 10.1038/s41598-017-03577-0

**Published:** 2017-06-12

**Authors:** Azra Ramezankhani, Farideh Bagherzadeh-Khiabani, Davood Khalili, Fereidoun Azizi, Farzad Hadaegh

**Affiliations:** 1 Prevention of Metabolic Disorders Research Center, Research Institute for Endocrine Science, Shahid Beheshti University of Medical Sciences, Tehran, Iran; 2 Department of Epidemiology, School of Public Health, Shahid Beheshti University of Medical Sciences, Tehran, Iran; 3 Endocrine Research Center, Research Institute for Endocrine Sciences, Shahid Beheshti University of Medical Sciences, Tehran, Iran

## Abstract

We identified risk patterns associated with incident coronary heart disease (CHD) using survival tree, and compared performance of survival tree versus Cox proportional hazards (Cox PH) in a cohort of Iranian adults. Data on 8,279 participants (3,741 men) aged ≥30 yr were used to analysis. Survival trees identified seven subgroups with different risk patterns using four [(age, non-HDL-C, fasting plasma glucose (FPG) and family history of diabetes] and five [(age, systolic blood pressure (SBP), non-HDL-C, FPG and family history of CVD] predictors in women and men, respectively. Additional risk factors were identified by Cox models which included: family history of CVD and waist circumference (in both genders); hip circumference, former smoking and using aspirin among men; diastolic blood pressure and lipid lowering drug among women. Survival trees and multivariate Cox models yielded comparable performance, as measured by integrated Brier score (IBS) and Harrell’s C-index on validation datasets; however, survival trees produced more parsimonious models with a minimum number of well recognized risk factors of CHD incidence, and identified important interactions between these factors which have important implications for intervention programs and improve clinical decision making.

## Introduction

According to the World Health Organization (WHO), in 2005, 30% of the total death was due to cardiovascular diseases (CVD), mainly heart disease and stroke^1^. It is estimated that by 2020, CVD will be the leading cause of mortality and morbidity worldwide, and developing countries will be the main contributors to this increase^2^. Greater burdens of CVD have been reported in Asian and Middle-Eastern regions^3^. It was reported that more than 40% of mortality in Tehran has been related to CVDs^4^, and about 20% of adults aged ≥30 years had symptoms or signs of CHD in Tehran^5^.

Traditional and conventional risk factors such as age, sex, hypertension, dyslipidemia, smoking and diabetes appear to be associated with CHD^6^. While vast amounts of studies have investigated the main effect of these risk factors^7, 8^, there is little information about how these risk factors interact with one another to produce different patterns of risk for CVDs. Identification of interactions between risk factors could provide insight into mechanisms of dependence, identify those subgroups at higher risk for the CVDs, and implement intervention programs that target high risk groups instead of all population^9^. Traditional regression models such as logistic or Cox proportional hazards (Cox PH) models are used to model the relationship between a set of risk factors and outcomes in medical domains. Cox PH has central importance in the analysis of censored failure time data. Although it has a semi-parametric form, but efficient inference can be made based on a partial likelihood approach^10^. Specifically, like linear and logistic regression, Cox PH is a linear method assumes that the logarithm of the hazard rate is linearly related to the covariates^10^; nevertheless, when nonlinear effects or complex interactions are involved, these methods may encounter problems^11–14^. On the other hand, assessment of interactions, using this model requires pre-specification of the interaction terms; as the number of variables in the model increases, the number of possible interactions become large^15^ and lead to a complicated model that can be difficult to fit and interpret^16^. For example, if we have 10 covariates, then there are (10 × 9)/2 = 45 two-way interaction terms for including into regression models. Furthermore, examining many interactions and including only the strongest in the regression model leads to biased estimation of the effects and overly optimistic performance estimates^12^.

Recursive partitioning or ‘decision trees’, the relatively recently developed methodology, are another class of nonparametric regressions which have been widely used in many fields^12, 13, 17–19^. These methods provide a very flexible framework without pre-specifying the interactions. Instead, they can assess interactions after tree are grown^12^. They make fewer modeling assumptions which can be used as an explorative method to partition objects in a data set into several homogenous groups with a similar outcome^12, 13^. Each partition is based on one predictor variable at a time; when numerous variables are tested at each recursive step, a tree selects the variable that most efficiently divides the subjects considering outcome likelihood. Hence, high-level interactions or nonlinear relationship between the predictors and the outcome variable can be captured automatically by decision trees which offer a new way to look at complex data sets^12, 13, 20^. Therefore, decision trees can be used as a supplemental tool when the conventional methods meet their limits. Moreover, by providing a flowchart-like graph, these methods are readily interpretable by users who are not too much familiar with statistical methods^20^. Survival trees are popular nonparametric alternatives to the Cox PH regression models which have been extended to survival analysis^12^. They can naturally group subjects according to their length of survival based on their covariates patterns^12, 14^. Several tree-based methods have been developed in survival setting which are extensions of the basic methods^14^. Conditional inference survival trees yields the smaller tree and avoid excessively growth of the tree compared to the other methods^21^. The aim of the present study was to use this type of survival trees to identify relative importance of factors contributing to the incidence of CHD, and detecting the subgroups with different survival functions based on related covariates (risk patterns). Second, we compared results of the Cox PH method with those produced by the survival tree analysis regarding important predictors of incidence of CHD and overall predictive performance they yield. We used the prospective cohort database from Tehran Lipid and Glucose Study (TLGS) for our analysis.

## Methods

### Study population

The TLGS study is an ongoing study being conducted among a representative sample of 15,005 people, aged ≥3 yr, residents of district-13 of Tehran^22^. Participants were recruited during 1999 to 2001 (phase 1). In the second phase (2002–2005), 3,551 new participants, aged ≥3 yr entered the study. We considered all subjects aged ≥30 yr from the first and second phases (n = 9,752) and excluded individuals with prevalent CVD at baselines (n = 605). We also excluded subjects that had participated only in baseline measurement, but did not respond to follow up surveys by the end of the study (20 March 2012) (n = 868), leaving us with 3,741 men and 4,538 women (90% of subjects were initially eligible for enrollment) (Supplementary Fig. S1). All experiments were performed in accordance with relevant guidelines and regulations. Informed consent was obtained from all participants and/or their legal guardians, and study protocol was approved by the ethical committee of the Research Institute for Endocrine Sciences of Shahid Beheshti University of Medical Sciences, Tehran, Iran.

### Clinical and laboratory measurements

At baselines, data were collected using interview, physical examination and laboratory measurements. Data included demographic data, past medical history, drug consumption, smoking behavior and physical activity. Blood pressure and anthropometrical measurements were collected during physical examinations. A blood sample was taken after 12 to 14 hours overnight fasting and total cholesterol (TC), high density lipoprotein cholesterol (HDL-C), triglycerides (TGs), fasting plasma glucose (FPG) and creatinine levels were measured using previously reported methods^22^. Non-HDL cholesterol (non-HDL-C) was defined as the difference between total and HDL-C. Estimated glomerular filtration rate (eGFR) was obtained using an equation derived from the modification of diet in the renal disease (MDRD) study^23^. Physical activity level was assessed with the Lipid Research Clinic (LRC) questionnaire in the first phase of the study. Due to the inexactness of LRC, it was substituted by the Modifiable Activity Questionnaire (MAQ) from the 2nd phase. This questionnaire measures all three forms of activities including leisure time, job, and household activities in the past year^22^.

### Definition of terms

Education level was categorized into 4 levels as illiterate, 1–9 years, 10–12 years and over 12 years of schooling. Marital status was categorized as single, married, widowed and divorced. A current smoker was defined as a person who smokes cigarettes or other smoking implements (water-pipes, pipes) daily or occasionally. Former smokers were defined as individuals who have smoked daily or occasionally and, those who have quit smoking. Passive smoking was defined as exposure to secondhand smoke in the home, at work, and other environments. A family history (FH) of premature CVD was considered as any experience of fatal or non-fatal myocardial infarction, stroke or sudden cardiac arrest in first-degree relatives, if it occurred before 55 years of age in male relatives (father, brother and son) and before 65 years of age in female relatives (mother, sister and daughter). Family history of diabetes (FHD^+^) was defined as having type 2 diabetes in first-degree relatives.

Low physical activity (Inactive) was defined as doing exercise or labor < three times a week or scores ≤600 MET (metabolic equivalent task)-minutes per week^24^. All Participants were also classified into two categories, based on whether or not participating in the life-style intervention.

For women, four additional variables were collected: (1) menstruation status with 3 categories (having normal or menstruating by taking medication, normal menopause and early menopause because of surgery or other reasons); (2) previous pregnancy history; (3) previous history of hypertensive pregnancies; and, (4) history of hyperglycemia in previous pregnancies.

### Definition of CHD outcome

Details of the outcome measurements have been described previously^22, 25^. Briefly, the follow up surveys were conducted annually by phone calls. A trained nurse asked each participant about any medical event leading to hospitalization during the past year, following which complementary data were collected by a trained physician. In the case of mortality, cause-of-death identified using death certificate, the forensic medicine report and if needed by verbal autopsy. Data collected were then evaluated by a committee of experts. In this study, outcomes were defined using ICD10 rubric I20–I25, and included definite myocardial infarction (MI) (with diagnostic ECG and biomarkers), probable MI (positive ECG findings plus cardiac symptoms or signs but biomarkers showing negative or equivocal results), unstable angina pectoris (new cardiac symptoms or changing symptom patterns and positive ECG findings with normal biomarkers), angiography proven CHD and any death due to CHD based on above criteria in hospital or sudden cardiac death from cardiac disease, in ≤1 hr after onset of symptoms, based on verbal autopsy documents outside of hospital.

Time to event was defined as time of censoring or having the CHD event, whichever occurred first. We censored individuals at the time of other causes of death, loss to follow up and being in the study until 20 March 2012 (end of study) without any CHD event.

### Statistical methods

#### Datasets

Two datasets were used in the present study; the first was for men (included 3,741 subjects, 29 predictor variables and 455 incident CHD events), and the second was for women (included 4,538 subjects, 33 predictors and 307 incident CHD events).

To deal with missing values (less than 6%) for some covariates (Supplementary Table S1) we used single imputation with the classification and regression tree (CART) method^26^ in SPSS modeler 14. After imputation, each dataset was divided into two parts, using stratified random sampling; one part (75%) for developing the models (train) and 25% for testing or validating the performance of the models (Supplementary Table S2).

#### Analysis methods

Baseline characteristics of participants were expressed in mean (standard deviation) and frequency (percentage) for continuous and categorical variables, respectively. Comparisons between men and women were performed using Student’s T-test or χ^2^ tests as appropriate. Crude incidence density rate of CHD and respective 95% confidence interval (CI) were calculated for each gender, by dividing the number of events to person-years at risk.

The multivariable analysis was done using conditional inference survival tree developed by Hothorn *et al*.^21^; it is an unbiased non-parametric class of regression trees, embedding tree-structured regression models into a well defined theory of conditional inference procedures^21^. Survival trees are created using a recursive partitioning algorithm. In step 1, the variable with the highest ability to separate survivors and non-survivors is found using p-values from permutation distributions. In step 2, a binary split (the best cut point) in the selected input variable is implemented; the cut points are selected based on log-rank statistics. Finally, in step 3, two previous steps recursively are repeated. The conditional inference tree does not use pruning; it uses stopping rules based on Bonferroni-adjusted p-values to determine tree size. A survival tree can naturally group subjects according to their survival time based on their covariates, and can hence automatically detect complex interactions between covariates without the need to specify them beforehand^21, 27^. We used all baseline variables for constructing survival tree models. The minimum criterion for node split was defined as p < 0.05, and the minimum subjects in terminal nodes were defined as sixty. The results are displayed as a single tree. Kaplan-Meier (KM) curves were constructed for each subgroup identified by the survival tree method. Also, we brought the identified tree structure into the Cox PH model by specifying one categorical variables with *k* level (*k* is the number of terminal nodes in survival trees) to estimate the hazard ratios (HR) of CHD events among the identified subgroups by considering the low risk group as reference. This idea has been reported previously for decision tree and logistic regression^12^.

In further analysis, the stepwise Cox PH regression model, using Akaike’s information criteria (AIC) as the model selection approach was implemented on the same data (train), outcome and covariates used for survival tree analysis to compare predictive performance of two survival tree and Cox regression approaches^28^.

#### Assessment and comparison of models performance

For the comparison of predictive performance of different models it is important that exactly the same data are used for the training all models^12, 20, 28^. Hence, in the present study all models were developed using the train datasets and performance of the models were evaluated using the test datasets. We used the integrated Brier score (IBS) on test datasets to evaluate performance of the survival tree and Cox PH models^29^. The IBS is a popular measure for the evaluation of overall performance (discrimination) of survival models^29^. The Brier score calculates the squared difference between true event status at given time and the predicted event status at that time; this score for a model can range from 0 for a perfect model to 0.25 for a non-informative model. IBS summarizes the prediction error over all times in the test dataset. Lower values indicate better predictive performance.

Also, Harrell’s C-index or concordance C^30^ was obtained as a measure of the general discrimination of the Cox models among training and testing samples. C-index is the fraction of pairs in the data, where the observation with the higher survival time has the higher probability of survival predicted by the model^31^. We used four packages of Party, Survival, ipred and survcomp from the R software version 3.2.5 (www.r-project.org) for our analysis. Two-tailed P values < 0.05 were considered statistically significant.

## Results

### Characteristic of participants

The study sample consisted of 3,741 men, aged 30–86 yr (mean age 47 ± 12.9) and 4,538 women, aged 30–88 yr (mean age 46 ± 11.6). After 37,808 and 48,090 person-years of follow-up, 455 and 307 first CHD events occurred in men and women, respectively. The crude incidence rate of CHD was calculated to be 12.1 (95% CI, 11.1–13.1) and 6.4 (95% CI, 5.7–7.1) per 1000 person-years in men and women, respectively. About 90% of population in both genders had complete data (complete cases), and range of missing values was 0.3–6.4% and 0.3–5.8% in men and female, respectively (Supplementary Table S1). Baseline characteristics of men and women, after imputation, on all independent variables are shown in Table 1.

**Table 1:** Baseline characteristics of participants after imputation of missing data, Tehran Lipid and Glucose Study (1999–2012).

Baseline characteristics	Men n = 3,741	Women n = 4,538	*P value
Continuous variables, mean (SD)			
***Age (years)***	47.5 (13.0)	46.1 (11.6)	<0.001
***Total length of stay in the city (years)***	38.8 (13.5)	37.8 (13.5)	<0.01
***Body mass index (BMI) (kg/m*** ^***2***^ ***)***	26.2 (3.9)	28.5 (4.7)	<0.001
***Waist circumference (cm)***	90.6 (10.8)	90.5 (12.0)	0.649
***Wrist circumference (cm)***	17.8 (0.9)	16.2 (1.0)	<0.001
***Hip circumference (cm)***	96.8 (6.9)	105.2 (9.3)	<0.001
***Fasting plasma glucose (mmol/L)***	5.52 (1.71)	5.58 (2.03)	0.112
***Triglyceride Levels (mmol/L)***	2.18 (1.95)	1.54 (1.24)	<0.001
***Total cholesterol (mmol/L)***	5.32 (1.09)	5.64 (1.21)	<0.001
***Non HDL cholesterol (mmol/L)***	4.35 (1.08)	4.49 (1.21)	<0.001
***HDL cholesterol (mmol/L)***	0.98 (0.24)	1.16 (0.29)	<0.001
***eGlomerular Filtration Rate (mL/min/1.73 m*** ^***2***^ ***)***	72.7 (11.4)	67.0 (10.8)	<0.001
Systolic blood pressure (mmHg)	120.7 (18.4)	120.7 (20.1)	0.912
Diastolic blood pressure (mmHg)	78.2 (11.0)	78.6 (10.8)	0.147
***Heart rate*** (beats/min)	75.1 (9.8)	81.7 (11.6)	<0.001
**Categorical variables, frequency (%)**			
***Education***			
level 1 (illiterate)	203 (5.4)	655 (14.4)	<0.001
level 2 (<9 years)	923 (24.7)	1497 (33.0)	
level 3 (10–12 years)	1936 (51.7)	2015 (44.4)	
level 4 (>12 years)	679 (18.2)	371 (8.2)	
***Marital status***			
Single	197 (5.3)	193 (4.3)	<0.001
Married	3509 (93.7)	3792 (83.5)	
Divorced	17 (0.5)	81 (1.8)	
Widowed	18 (0.5)	472 (10.4)	
***Family history of premature cardiovascular diseases in female relatives***	280 (7.5)	377 (8.3)	0.090
***Family history of premature cardiovascular diseases in male relatives***	271 (7.2)	503 (11.1)	<0.001
***Family history of diabetes in first-degree relatives***	962 (40.4)	1417 (59.6)	<0.001
***Physical activity levels***			
Inactive	2778 (74.3)	3283 (72.3)	0.050
***Exposed to second hand smoke at home or work***	1054 (28.2)	936 (47.0)	<0.001
***Former cigarette smoking***	1622 (43.4)	305 (6.7)	<0.001
***Current cigarette smoking***	1176 (31.4)	216 (4.8)	<0.001
**Use of blood lipid lowering drugs**	77 (2.1)	217 (4.8)	<0.001
**Use of blood glucose lowering drugs drugs, n (%)**	125 (3.3)	240 (5.3)	<0.001
**Use of anti hypertensive drugs drugs, n (%)**	149 (4.0)	465 (10.2)	<0.001
***Use of aspirin***	405 (10.8)	470 (10.4)	0.490
***Participating in the life-style intervention group***	1677 (44.8)	2098 (46.2)	0.202
***Menstruation status***			
Normal Menstruation	—	2789 (61.5)	—
Menopause	—	1239 (27.3)	—
Early Menopause	—	510 (11.2)	—
***Previous pregnancy history***	—	4149 (91.4)	—
***Previous history of hypertensive pregnancies***	—	292 (6.4)	—
***History of hyperglycemia in previous pregnancies***	—	62 (1.4)	—

^*^P-values for difference between groups were calculated with Student’s T-test or χ2 tests for continuous and categorical variables, respectively.

Women had higher mean for BMI, hip circumference, TC, non HDL-C, HDL-C and heart rate; in addition, they had higher frequencies of FH of premature CVD in male relatives and FHD in first-degree relatives. They were also less educated, physically more inactive, and had lower smoking rates. Survival curve has been shown in Supplementary Fig. S2 for men and women (Log-Rank χ^2^ = 79.3, P < 0.001 for equality of survivor functions).

### Survival tree model for men

**Figure 1:**
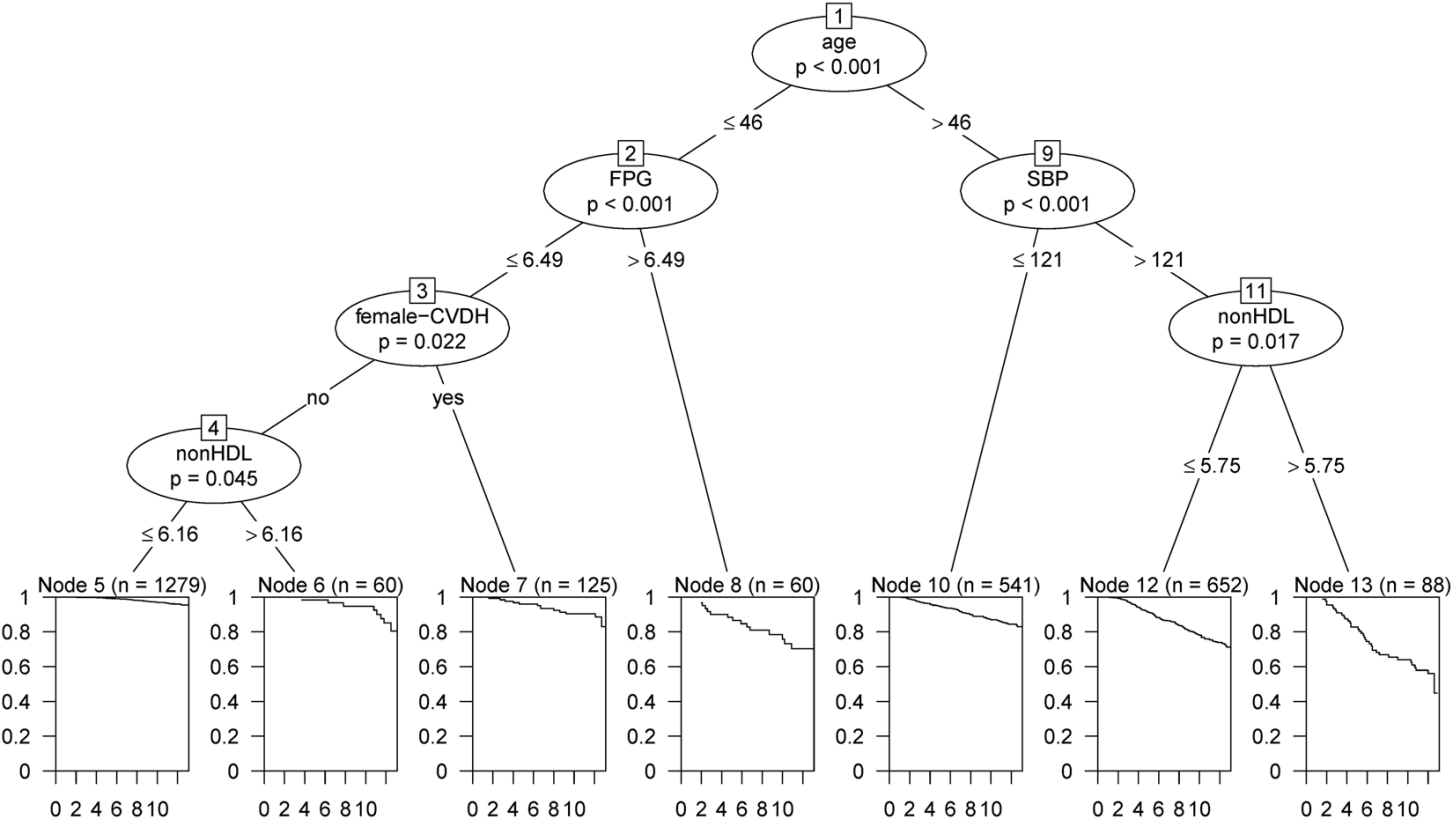
Survival tree model for incidence of CHD events in men and the distribution of survival times in the terminal nodes, Tehran Lipid and Glucose Study (1999–2012). At each level, the most significant split based on log-rank (LR) and permutation P value is shown. **SBP**: Systolic blood pressure; **FPG**: Fasting plasma glucose; **female-CVDH**: Family history of CVD in female relatives

The fitted tree for men (Fig. 1) showed that age, SBP, FPG, non-HDL-C and history of premature CVD in female relatives were the predominant factors related to survival probability. Each path from a root node through terminal nodes is a pattern of covariates which can distinguish a group according to their survival function. Starting with the root node, which included all training data, the tree first divided the participants into two groups based on age, with a cut off of 46 yr. On the right side of the tree, the subgroup aged >46 yr was split by SBP with cut off of 121 mmHg; the subgroup with SBP ≥121 mmHg was subsequently split by non-HDL-C with cut off of 5.75 mmol/L. Among the three subgroups extracted from the right branch of the tree (terminal nodes 10, 12 and 13), those aged >46 yr, with SBP >121 mmHg and non-HDL-C >5.75 mmol/L exhibited the lowest survival probability. It was also observed that those aged >46 yr, with SBP ≤121 mmol/L had better survival, compared to two groups with SBP >121 mmHg (nodes 12 and 13). Generally, covariate patterns in the right side of tree show that SBP and non-HDL-C are the most important factors related to CHD incidence among men aged >46 yr. On the left side of the tree, the subgroup aged ≤46 yr was split by FPG level with cut off of 6.49 mmol/L; a group of men with FPG ≤6.49 mmol/L was split by history of CHD in female relatives, then, those with positive history of CHD was subsequently split by non-HDL-C with cut off of 6.16 mmol/L. Among subgroups with age ≤46 yr, the group with FPG ≤6.49 mmol/L, negative history of premature CVD and non-HDL-C ≤6.16 mmol/L exhibited higher survival probability; whereas, those with FPG >6.49 mmol/L had lowest survival compared to the other three groups. In fact, covariate patterns in the left side of tree show that FPG, FH of premature CVD and non-HDL-C are the most important factors in relation with CHD incidence among men aged ≤46 yr.

The KM survival functions of seven subgroups identified by the survival tree model, has been shown in Supplementary Fig. S3. The KM plot of the survival function for the whole train data has been defined as reference. As seen, the curves of nodes 13, 8 and 12 are much lower than the reference curve, showing that they cover high risk groups for the CHD event. The symmetric remark can be made for nodes 5, 6, and 7, which have a survival probability above the reference curve which are due to low-risk populations.

A Cox PH model was fitted on the train dataset using a categorical variable with 7 levels; each level identified a risk group or terminal node (nodes 5 to 13). Node 5 was considered as the reference group, and HRs for other nodes were compared to it. Node 13 had the highest risk for CHD event (HR, 15.49; 95% CI, 9.97–24.08; p < 0.001). Table 2 shows the pattern of each terminal node of the survival tree and its related HRs.

**Table 2:** Cox analyses of patterns identified using survival tree in the male population, Tehran Lipid and Glucose Study (1999–2012).

Nodes	Number of cases/number of events	Pattern description	HR (95% CI)	P value
5	1, 279/44	Age ≤46 yr, FPG ≤6.49 mmol/L, negative history of CHD in female relatives, non-HDL-C ≤6.16 mmol/L	Reference category	
6	60/8	Age ≤46 yr, FPG ≤6.49 mmol/L, negative history of CHD in female relatives, non-HDL-C >6.16 mmol/L	3.79 (1.79–8.06)	<0.001
7	125/13	Age ≤46 yr, FPG ≤6.49 mmol/L, positive history of CHD in female relatives	3.11 (1.67–5.77)	<0.001
8	60/15	Age ≤46 yr, FPG >6.49 mmol/L	8.88 (4.94–15.96)	<0.001
10	541/75	Age >46 yr, SBP ≤121 mmol/L	4.28 (2.95–6.20)	<0.001
12	652/150	Age >46 yr, SBP >121 mmol/L, non-HDL-C ≤5.75 mmol/L	7.61 (5.44–10.65)	<0.001
13	88/36	Age >46 yr, SBP >121 mmol/L, non-HDL-C >5.75 mmol/L	15.49 (9.97–24.08)	<0.001

**Nodes**: Terminal nodes number of survival tree for men; **CI:** Confidence intervals; **CHD**: Coronary heart disease; **FPG**: Fasting plasma glucose; **HR**: Hazard ratio; **SBP**: Systolic blood pressure.

### Predictors of incident CHD in men identified by the Cox PH

In the multivariate Cox PH model, age, WC, FPG, non-HDL-C, SBP, FH of premature CVD in female relatives, past smoking and use of aspirin were positively, and hip circumference was negatively related to the incidence of CHD in men (Table 3).

**Table 3:** Multivariate Cox regression analysis of factors associated with CHD incidence in male population (n = 2,805), Tehran Lipid and Glucose Study (1999–2012).

Variables	HRs (95% CI)	P value
***Age (years)***	1.04 (1.03–1.05)	<0.001
***Waist circumference (cm)***	1.02 (1.01–1.04)	0.002
***Hip circumference (cm)***	0.97 (0.94–0.99)	0.010
***Fasting plasma glucose (mmol/L)***	1.10 (1.06–1.14)	<0.001
***Non-HDL cholesterol (mmol/L)***	1.26 (1.16–1.38)	<0.001
***Systolic blood pressure (mmHg)***	1.01 (1.01–1.02)	<0.001
***Family history of premature cardiovascular diseases in female relatives***		
No	Reference	
Yes	1.50 (1.21–1.86)	<0.001
***Former cigarette smoking***		
No	Reference	
Yes	1.83 (1.30–2.59)	<0.001
***Use of aspirin drugs***		
No	Reference	
Yes	1.45 (1.09–1.92)	0.008

Cox proportional hazard regression model with stepwise selection method was used to calculate hazard ratios (HRs) and 95% confidence interval (CI), and Akaike information criteria (AIC) was used for model selection approach.

### Survival tree for women

**Figure 2:**
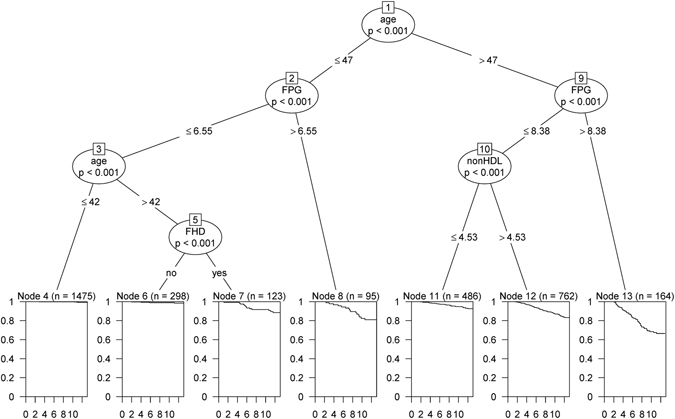
Survival tree model for incidence of CHD events in women and the distribution of survival times in the terminal nodes, Tehran Lipid and Glucose Study (1999–2012). At each level, the most significant split based on log-rank (LR) and permutation P value is shown. **FHD**: Family history of diabetes; **FPG**: Fasting plasma glucose.

The survival tree for women is shown in Fig. 2. Initially the participants were split by age with the best cutoff of 47 yr. On the right side of the tree, the group aged >47 yr was split by FPG with cutoff of 8.38 mmol/L; then, the group with FPG ≤8.38 mmol/L was split by non-HDL-C with cutoff of 4.53 mmol/L. Therefore, three groups (nodes 11, 12 and 13) were ultimately defined on the right side of the tree. KM curves show that among subgroups aged >47 yr, those with FPG >8.38 mmol/L has lowest survival probability; while, women with FPG ≤8.38 mmol/L and non-HDL-C ≤4.53 mmol/L has higher survival, compared to the other two groups (nodes 12 and 13). Generally, results of survival tree showed that among women aged >47 yr, FPG and non-HDL-C had the most predictive role for incidence of CHD. On the left side of the tree, the group aged ≤47 yr was split by FPG level with cutoff of 6.55 mmol/L; then, the subgroup with FPG ≤6.55 mmol/L was split by age (cutoff of 42 yr). Finally, those women aged 42–47 yr was split by FHD. Therefore, four subgroups were identified which node 4 with the covariate pattern of (age ≤42 yr and FPG ≤6.55 mmol/L) had the highest survival probability; while, those aged ≤47 yr and FPG >6.55 mmol/L had the lowest. In short, among women aged ≤47 yr, FPG and FHD^+^ were the most important factors in relation with CHD incidence. In Supplementary Fig. S4, we have illustrated the KM plot for survival functions of seven subgroups identified by the tree model. The KM plot shows that nodes 13, 8 and 12, cover high risk groups for the CHD events. In contrast, nodes 4 and 6 define low-risk populations. Table 4 shows the results of the Cox PH model fitted on the train dataset. Considering node 4 as the reference category, node 13 had the highest risk for CHD events (HR, 56.54; 95% CI, 28.63–111.67; p < 0.001).

**Table 4:** Cox analyses of patterns identified using survival tree in the female population, Tehran Lipid and Glucose Study (1999–2012).

Nodes	Number of cases/number of events	Pattern description	HR (95% CI)	P value
4	1, 475/10	Age ≤42 yr, FPG ≤6.55 mmol/L	Reference category	
6	298/4	Age (42–47) yr, FPG ≤6.55 mmol/L, without FHD	2.03 (0.636–6.47)	0.23
7	123/13	Age (42–47) yr, FPG ≤6.55 mmol/L, with FHD	16.21(7.110–36.98)	<0.001
8	95/15	Age ≤47 yr, FPG >6.55 mmol/L	26.51 (11.91–59.02)	<0.001
11	486/28	Age >47 yr, FPG ≤8.38 mmol/L, non-HDL ≤4.53 mmol/L	9.40 (4.564–19.34)	<0.001
12	762/111	Age >47 yr, FPG ≤8.38 mmol/L, non-HDL >4.53 mmol/L	23.02 (12.05–43.96)	<0.001
13	164/49	Age >47 yr, FPG >8.38 mmol/L,	56.54 (28.63–111.67)	<0.001

**Nodes**: Terminal nodes number of survival tree for women; **CI:** Confidence intervals; **FPG:** Fasting plasma glucose**; FHD**: Family history of diabetes; **HR**: Hazard ratio.

### Predictors of incident CHD in women identified by the Cox PH

Results of multivariate Cox PH model in women showed that, age, WC, FPG, non-HDL-C, DBP, FH of premature CVD in male relatives and using lipid lowering drugs were positively related to the incidence of CHD in women (Table 5).

**Table 5:** Multivariate Cox regression analysis of factors associated with CHD incidence in female population (n = 3,403), Tehran Lipid and Glucose Study (1999–2012).

Variables	HRs (95% CI)	P value
***Age (years)***	1.05 (1.04–1.07)	<0.001
***Waist circumference (cm)***	1.02 (1.01–1.03)	0.003
***Fasting plasma glucose (mmol/L)***	1.13 (1.09–1.17)	<0.001
***Non-HDL cholesterol (mmol/L)***	1.21 (1.09–1.33)	<0.001
***Diastolic blood pressure (mmHg)***	1.01 (1.01–1.03	0.013
***Family history of premature cardiovascular diseases in male relatives***		
No	Reference	
Yes	1.98 (1.43–2.72)	<0.001
***Use of lipid lowering drugs***		
No	Reference	
Yes	1.58 (1.08–2.31)	0.016

Cox proportional hazard regression model with stepwise selection method was used to calculate hazard ratios (HRs) and 95% confidence interval (CI), and Akaike information criteria (AIC) was used for model selection approach.

### Performances of the survival tree and Cox PH models

The IBS and C index for survival trees and Cox models obtained among train and test samples are shown in Table 6. The IBS values from the survival tree and Cox models were very close. However, in women, the C index for the Cox PH on nodes was slightly higher than the C index for the Multivariate Cox PH (0.838 vs. 0.806); in contrast, opposite results was observed in men (0.740 vs. 0.771).

**Table 6:** Performance of the Cox PH and survival tree models among train and test samples; Tehran Lipid and Glucose Study (1999–2012).

	IBS		C index	
	Test data	Train data	Test data	Train data
**Men**				
Survival tree	0.060	0.061	—	—
Multivariate Cox PH	0.056	0.057	0.771	0.767 (0.735–0.799)
Cox PH on nodes	0.056	0.057	0.740	0.731 (0.701–0.761)
**Women**				
Survival tree	0.032	0.033	—	—
Multivariate Cox PH	0.033	0.033	0.806	0.827 (0.789–0.865)
Cox PH on nodes	0.030	0.031	0.838	0.805 (0.769–0.841)

**IBS:** Integrated Brier score; is defined as the squared difference between true event status at given time and the predicted event status at that time. **C index:** Harrell’s C-index; is defined as the fraction of pairs in the data, where the observation with the higher survival time has the higher probability of survival predicted by the model. Number of subjects in train data for men = 2,805; Number of subjects in test data for men = 936. Number of subjects in train data for women = 3,403; Number of subjects in test data for men = 1,135.

## Discussion

This is the first epidemiological investigation to have ever compared survival tree versus Cox regression to predict CHD incidence in a large population. The present population based study, determined the 12-year incidence of CHD as well as risk factors for CHD and interactions between those risk factors. The survival tree model identified seven groups among men only by five factors (age, FPG, SBP, non-HDL-C and history of CVD in female relatives). In women, the survival tree extracted seven groups by four factors i.e. age, FPG, FHD and non-HDL-C.

Consistent with previous research, the multivariate Cox PH models identified: age^32, 33^, WC^34, 35^, FPG^36, 37^ and non-HDL-C^38–40^ as predictors of CHD in both genders. Specifically, SBP^37^, hip circumference^41, 42^, FH of premature CVD in female relatives^34^, past smoking^34, 37^ and use of aspirin^43^ were significant predictors for incident CHD in men. However, in women, DBP^34^, FH of premature CVD in male relatives^34^ and use of lipid lowering drugs were significantly associated with incidence of CHD. Generally, both Cox PH and survival tree models showed good performance in women compared to men.

Both sets of analyses in the present study revealed the importance of age in predicting CHD. Existing data clearly shows that age, is the major risk factor for CVD^32, 33^. According to existing studies, ages 45 and 55 years or higher have been known as high risk ages in men and women, respectively^8^. Our previous study showed that in a multivariable adjusted Cox PH model, HRs were 3.9 and 2.7 for men aged >45 yr and in women aged >55 yr for incidence of CHD, respectively^7^. In the present study, Survival tree analysis determined almost identical cut points of 46 and 47 yr for men and women, respectively. Our finding suggests that the threshold considered for definition of high risk age in women (55 yr) might be even lower among Iranian women, for incidence of CVD. This is consistent with finding from our recent study that showed women had high incidence rate of premature CVDs after the age of 45 years^44^.

The results indicated that SBP was the most important predictor among men aged >46 yr; those with age >46 yr and SBP ≤121 mmHg had a marked decreased risk of CHD events compared to those men with age >46 yr and SBP >121 mmHg. However, the former group had about 4-fold higher risk of CHD compared to reference group. This result confirms the important role of aging in incidence of CHD; because, even normotensive men aged >46 yr had significant increased risk of CHD independent of other predictors.

In 2003, a new category of blood pressure was defined; SBP between 120–139 mmHg or DBP between 80–89 mmHg were classified as prehypertension^45^. A meta-analysis with data of 18 prospective cohort studies showed that prehypertension significantly increased the risk of CHD in high range prehypertension, but not in low-range prehypertensive populations^46^. The present study showed that SBP >121 mmHg were associated with higher risk of CHD in men aged >46 yr, especially when co-occurs with non-HDL-C >5.75 mmol/L.

Several studies have shown that non-HDL-C level is an independent risk predictor for CHD incidence^39, 40^. Some investigators have suggested that non-HDL-C may be superior to LDL-C as a predictor of CHD^38^. Using data from the Framingham Heart Study, Liu *et al*. showed a significant association between incidence of CHD and non-HDL-C; they categorized non-HDL-C into three levels. The HR for CHD was 2.21 and 2.34 for non-HDL-C ≥ 4.9 compared to non-HDL-C ≤4.1, in men and women, respectively^47^.

Considering the high prevalence of prehypertension and hypercholesterolemia among Iranian population^48^, our finding may represent an important adjunctive strategy to men aged >46 for long-term CHD prevention using lifestyles modification, adherence to therapy or modify treatment goals.

For younger men (≤46 yr), the pattern of FPG < 6.49 mmol/L, negative history of CVD in female relatives and non-HDL-C < 6.16 mmol/L identified a group with lowest risk for CHD (reference group). Despite data have reporting that type 2 diabetes increases the risk of CHD in both genders, the association between impaired fasting glucose (IFG) (5.5 ≤ FPG < 6.49 mmol/L)^49^ and risk of CVD is unclear^36^. A recently published meta-analysis reported that the presence of IFG was significantly associated with future risk of CHD in both genders; their results showed the significant risk of CHD even with FPG level of 5.5 mmol/L^36^. We showed that FPG >6.49 mmol/L, which covers IFG and diabetes, is the most important risk factor for CHD in younger men (<46 yr). In our previous study, we estimated that over 4% of Iranian populations develop pre-diabetes each year^50^, emphasizing the importance of implementing interventions in a large population for preventing CHD.

The survival tree also showed different interactions between FPG level, FH of CVD in female relatives and non-HDL-C in younger men. Numerous population-based studies demonstrated FH of premature CVD to be an established independent risk factor for CHD risk^51^; there is also evidences showing that positive FH of CVD in young population is the strongest clinical predictor of future CHD events, although, the association in women is controversial^52^. We observed a moderate risk for CHD in young men (<46 yr) with a positive history of premature CVD in their female relatives.

Results of the survival tree in women showed that FPG levels >8.38 mmol/L was the most important risk factors in older age (>47 yr). As FPG level of 8.38 mmol/L translates into a HbA_1_C levels of >7% which indicates poor glucose control^53^, our finding showed that diabetic women aged ≥47 yr with poor control of glucose exhibited the higher risk for CHD among the seven groups identified.

Type 2 diabetes has long been known as a risk factor for CHD, an association suggested being stronger in women than in men^54^. Three meta-analyses on this topic have documented conflicting results^54^. Some studies suggest that compared to men, diabetes may induce a more unfavorable cardiovascular risk profile among women^55^. In the present study, we found that women aged >47 yr with diabetes had significantly higher levels of total cholesterol (6.4 vs. 5.7 mmol/L) and non-HDL-C (5.2 vs. 4.7 mmol/L) than diabetic men aged >46 yr (data not shown).

Further exploration of the survival tree showed that non-HDL-C was a very close competitor for FPG as a splitter for women aged >47 yr. In fact, higher values of FPG (>8.38 mmol/L) was the most important factors predicting CHD risk among women aged ≥47 yr; however, in lower values of FPG (<8.38 mmol/L), non-HDL-C is the next important factor. Several studies suggest that non-HDL-C is particularly useful in predicting CVD risk in patients with diabetes^38^. Our results showed that in women this association depended on the FPG level. Our findings showed that, compared to other factors, among younger women aged ≤47 yr, FPG >6.55 mmol/L leads to highest risk of CHD, a finding suggesting that pre-diabetes and diabetes, is the most important risk factor among women aged ≤47 years. Women aged ≤42 yr and FPG level of ≤6.55 mmol/L had the lowest risk for CHD; with increasing age (42–47 yr), FHD^+^ leads to moderately increased risk for CHD events. The importance of FHD^+^ as a risk factor for CVD remains controversial^56^.

Almost all covariates identified by survival tree models were also significant predictors of CHD in the multivariate Cox models; however, Cox models identified some extra covariates that were not observed in the survival trees. We should note that in survival tree, not all the covariates entering the program will be appear in the final model; but, only the covariates that best split the data and meet particular criteria (permutation P value), are selected^57^. As Tables 3 and 5 show, Cox models identified only the main effect of the covariates but failed to show interaction between variables which has been identified during survival tree-growing process. Although we can include interaction terms in the Cox models in order to estimate the joint effect of covariates on the survival time, but in this case the statistical modeling becomes rather large which will over fit the data and the interpretation of the results will be quite complicated.

Survival trees and multivariate Cox models yielded comparable IBS on test datasets; however, survival trees produced more parsimonious models than the multivariate Cox models; they provided accurate prognostic tool for the estimation of survival functions using only 4 and 5 predictors in women and men, and identified potentially important interactions that is readily interpretable in terms of dividing the population into groups that are at higher or lower risk for CHD.

Survival patterns can be represented by simple if-then rules, which, unlike other machine learning methods such as neural network^58, 59^, can be easily understood by clinicians; also, they produce research hypotheses for further investigation by the experts.

Our results suggest that survival tree analysis can be viewed as complementary analytic approaches when the research question is exploration of conditional information and detecting some homogeneous group of individuals with different survival probability^57^. In fact, researchers can gain additional insights on a phenomenon by using survival tree analysis.

The strengths of this study included the use of data from a population based study (TLGS) with a relatively long term follow-up. Compared to our previous studies, we included more covariates to assess the true relationship between these variables and CHD outcomes. Moreover, this study benefits from a small number of lost to follow-up. As limitation, this study has been conducted on a representative sample of residents of Tehran, the capital of Iran, so our results cannot be directly extrapolated to other racial/ethnic populations.

## Conclusions

The performance of survival trees and multivariate Cox models on test datasets were substantially similar as assessed by IBS. However, survival trees produced more parsimonious models with a minimum number of classic and well recognized risk factors of CHD incidence and identified potentially important interactions between those factors. Our results also highlight the complementary nature of survival tree and Cox PH regression to providing the prediction models for small subgroups and overall population, respectively.

## Electronic supplementary material

data-supplement
