# Supplementary material for: A new look at risk patterns related to coronary heart disease incidence using survival tree analysis: 12 Years Longitudinal Study
Source: Sci Rep. 2017 Jun 12;7:3237. doi: 10.1038/s41598-017-03577-0 (PMC5468345; doi:10.1038/s41598-017-03577-0)
Supplement: Supplementary file 1 — data-supplement [file 41598_2017_3577_MOESM1_ESM.pdf]

A New Look at Risk Patterns Related to Coronary Heart Disease Incidence Using Survival Tree Analysis: 12 Years Longitudinal Study

Azra Ramezankhani, Farideh Bagherzadeh-Khiabani, Davood Khalili, Fereidoun Azizi, Farzad Hadaegh

Supplemental materials

Supplementary Table S1 Frequency of missing values for each variable before imputation, Tehran Lipid and Glucose Study (1999-2012)

Variables	Men n=3741	Women n=4538
	Frequency (%)	Frequency (%)
Age (years)	0	0
Total length of stay in the city (years)	11(0.3)	14 (0.3)
Body mass index (BMI) (kg/ m^2)	108 (2.9)	145 (3.2)
Waist circumference (cm)	108 (2.9)	163 (3.6)
Wrist circumference (cm)	108 (2.9)	127 (2.8)
Hip circumference (cm)	108 (2.9)	163 (3.6)
Fasting plasma glucose (mmol/L)	153 (4.1)	77 (1.7)
Triglyceride Levels (mmol/L)	153 (4.1)	73 (1.6)
Total cholesterol (mmol/L)	150 (4.0)	73 (1.6)
Non HDL cholesterol (mmol/L)	161 (4.3)	77 (1.7)
HDL cholesterol (mmol/L)	157 (4.2)	77 (1.7)
eGlomerular Filtration Rate (mL/min/1.73 m^2)	153 (4.1)	73 (1.6)
Systolic blood pressure (mmHg)	116 (3.1)	68 (1.5)
Diastolic blood pressure (mmHg)	116 (3.1)	68 (1.5)
Heart rate (beats/min)	116 (3.1)	73 (1.6)
Education	0	0
Marital status	0	0
Family history of premature cardiovascular diseases in female relatives	0	0
Family history of premature cardiovascular diseases in male relatives	0	0
Family history of diabetes in first-degree relatives	0	0
Physical activity levels	239 (6.4)	263 (5.8)
Exposed to second hand smoke at home or work	0	0
Former cigarette smoking	82 (2.2)	82 (1.8)
Current cigarette smoking	82 (2.2)	82 (1.8)
Use of blood lipid lowering drugs	0	0
Use of blood glucose lowering drugs drugs, n (%)	0	0
Use of anti hypertensive drugs drugs, n (%)	0	0
Use of aspirin	0	0
Participating in the life-style intervention group	0	0
Menstruation status	-	0
Previous pregnancy history	-	0
Previous history of hypertensive pregnancies	-	0
History of hyperglycemia in previous pregnancies	-	0

Supplementary Table S2 Characteristics of the train and test datasets, Tehran Lipid and Glucose Study (1999-2012)

Dataset		Number of cases	Number of CHD events	Ratio of event in dataset
**Men**	Train	2805	341	0.12
	Test (Validation)	936	114	0.12
**Women**	Train	3403	230	0.06
	Test (Validation)	1135	77	0.06

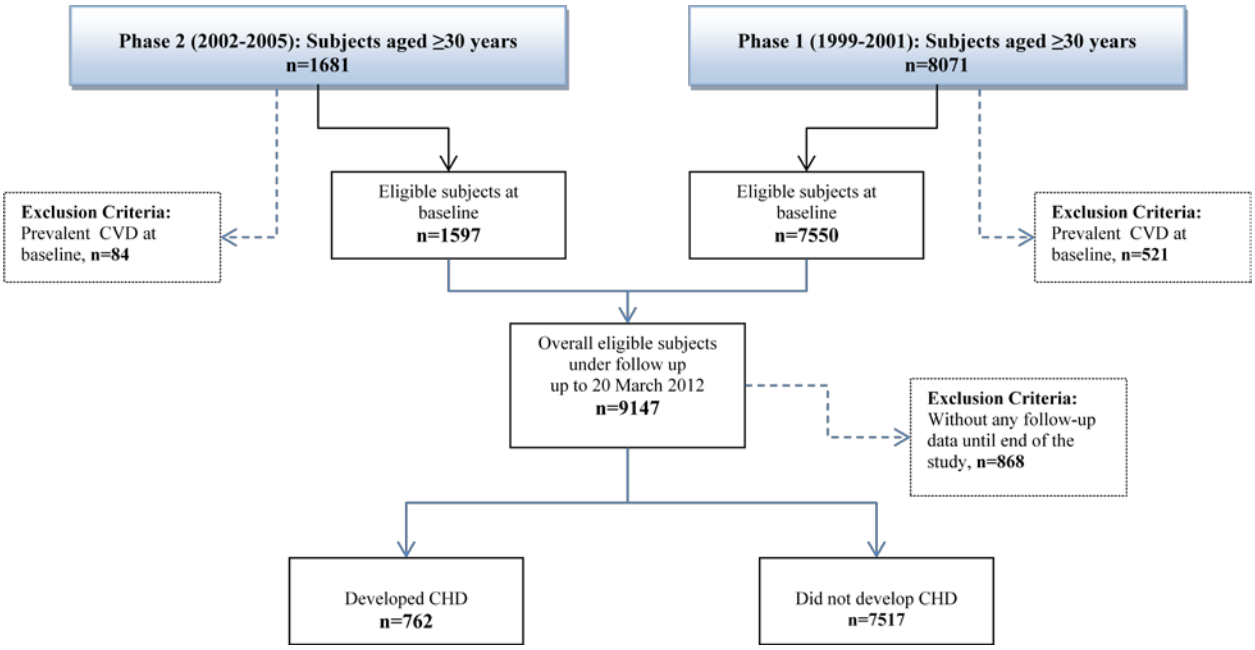

Supplementary Figure S1. Flow diagram for the selection of study subjects, Tehran Lipid and Glucose Study (1999-2012)

Supplementary Figure S2

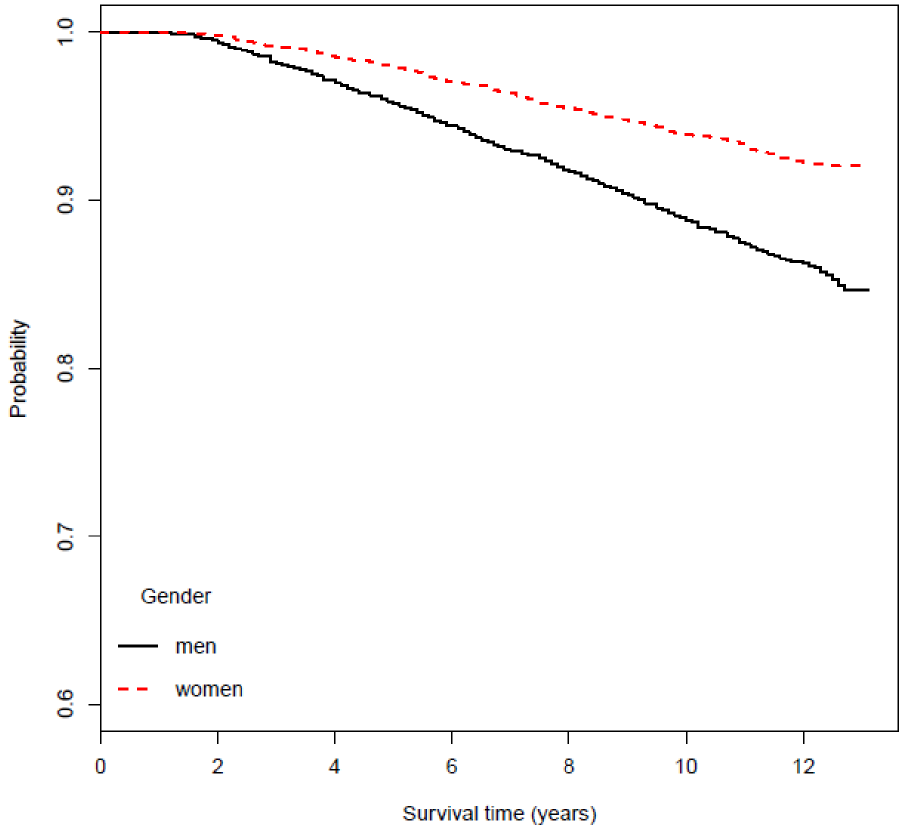

Supplementary Figure S2. Kaplan-Meier survival curves across genders, Tehran Lipid and Glucose Study (1999-2012)

Supplementary Figure S3

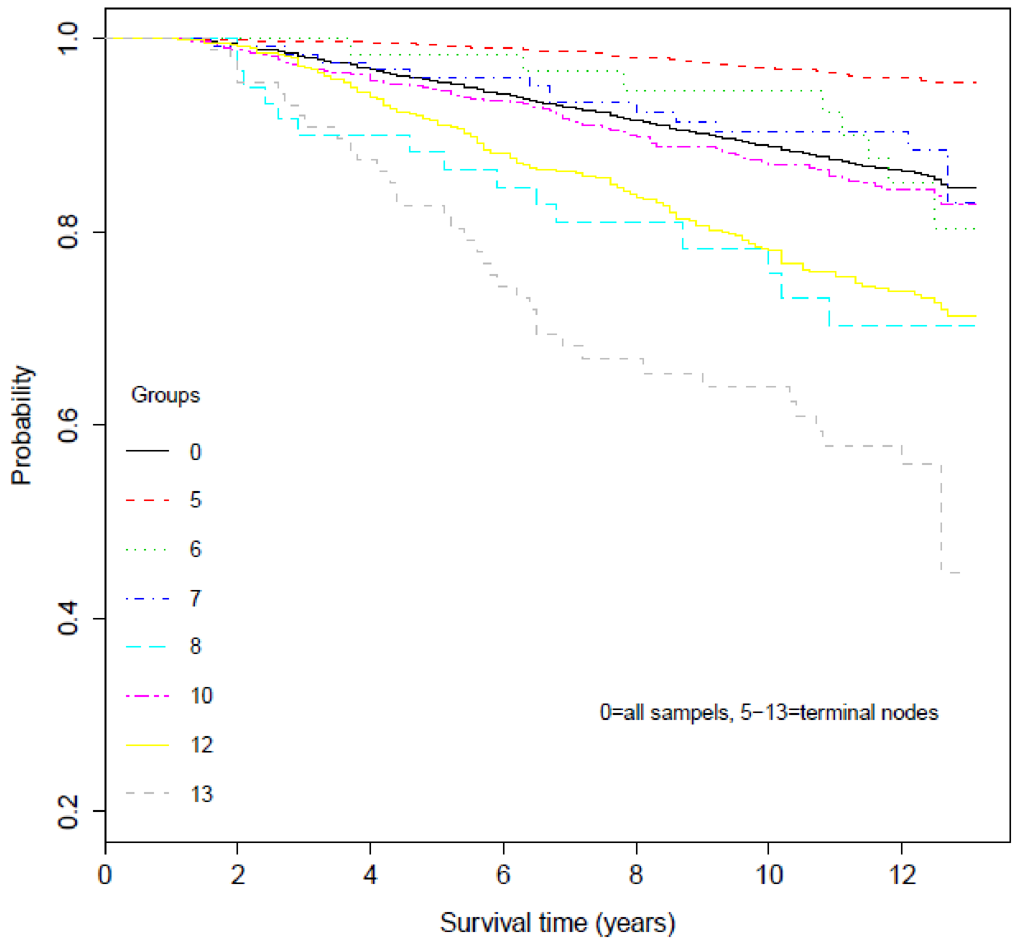

Supplementary Figure S3. Kaplan-Meier curves for incidence of CHD event in seven groups of men identified using the survival tree model. Tehran Lipid and Glucose Study (1999-2012). The solid line shows the KM plot for whole population in train data.

Supplementary Figure S4

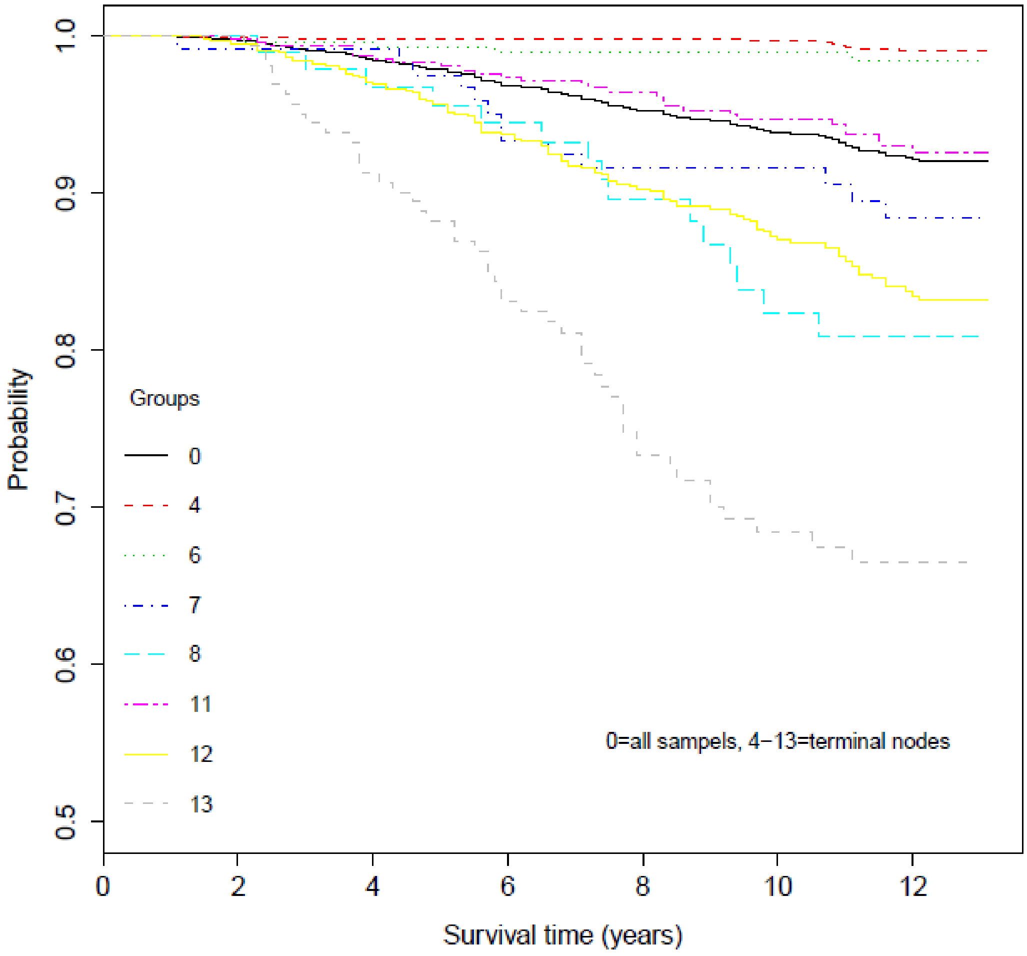

Supplementary Figure S4. Kaplan-Meier curves for incidence of CHD event in seven groups of women identified using the survival tree model, Tehran Lipid and Glucose Study (1999-2012). The solid line shows the KM plot for whole population in train data.
